# New York Odontological Society

**Published:** 1893-08

**Authors:** 

**Affiliations:** New York Odontological Society


### Reports of Society Meetings.

### NEW YORK ODONTOLOGICAL SOCIETY.

A REGULAR meeting of the New York Odontological Society was held on Tuesday evening, May 16, 1893, at the New York Academy of Medicine, No. 17 West Forty-third Street, New York City. Dr. Woodward, the President, being absent, the chair was occupied by Dr. Brockway.

The minutes of the previous meeting were read and approved.

### INCIDENTS OF PRACTICE AND COMMUNICATIONS.

Dr. Benjamin Lord read a short paper on the subject of combination fillings.

(For Dr. Lord's paper, see page 570.)

*Dr. Davenport.*—For some time I have intended saying a few words about the combination of gold with zinc phosphate, but forgot it until it was brought to my mind this evening. We remember with pleasure that Dr. Clapp, of Boston, gave us a little talk some time ago, and emphasized by a beautiful exhibition of specimens his different methods. I do not intend to contradict what Dr. Clapp said, for he has proved that in his hands the combination of gold and zinc phosphate, as well as the other combinations, is successful, but I do think the method should be used with extreme care. It was only a few days after that meeting that I had occasion to examine the teeth of two sisters who had but a few months before been discharged "cured" by their dentist, he having made many large gold fillings for them. Considerable pain of an undefined character coming to the young ladies, they went back several times, and being told that nothing could possibly be the matter, had at last been sent to me with the hope that I could discover the cause of the trouble. Several large gold fillings showed by the color about them that something was wrong, and as a test I decided to remove one of them. Drilling through the gold, the burr suddenly entered what appeared to be a deep open cavity, and a little later, cutting out more of the gold, I found that there was a base of zinc phosphate, which had evidently been packed very hurriedly, and, as it seemed to me, with the idea of placing gold upon and into it before the cement had hardened. These large cavities had been filled, most of them, in that way, and almost without exception were giving trouble. I thought at the time Dr. Clapp was telling us about his method that, in my hands at least, the proper placing of the varying zinc phosphates which are furnished us would be exceedingly doubtful if I were to pack gold into it before it had fully hardened, as, of course, must be done if the adhesion of the two materials is to be obtained.

*Dr. Howe.*—I would like to ask Dr. Lord whether, in referring to the proportions of tin and gold, he means them to be considered by weight?

*Dr. Lord.*—No, not by weight, but by the width of the strip of tin and the width of the strip of gold. I get the proportions in that way, then lay the tin on the gold and fold the gold over and over, which keeps the tin inside the gold.

*Dr. Howe.*—Will Dr. Lord tell us whether he refers to the same numbers of gold-foil and tin-foil, as, for instance, No. 4 gold and No. 4 tin?

*Dr. Lord.*—I use No. 5 gold, and tin, I think, of about the same number; but I always use No. 5 gold, both cohesive and non-cohesive.

*Dr. Howe.*—Mr. President, I have a suggestion to make in the line of practice. I will begin by recalling to your minds the fact that some years ago it was advocated by some to use pepsin in some form in the pulp-canals of pulpless teeth to digest devitalized tissue. The idea seemed to be a good one, for there is often material there which, if it could be chemically changed by some digestive process, would be more easily removed. Recently the necessity of removing partly disintegrated pulp-tissue from canals that were rather difficult of access recalled that former practice to my mind, and also the fact that a vegetable digestive has recently been presented to the medical profession called Papoid, made by Johnson & Johnson. I have used papoid in the pulp-cavities of teeth a few times with what appeared to be favorable results. I speak of it merely as a suggestion, that it may be experimented with; I do not intend to make an endorsement of it. I remember distinctly how many things we have known that were grand successes for a brief time.

*Dr. Ives.*—I think Dr. Howe has struck a good idea. I have had some experience with papoid. Its great characteristic is that it acts equally well with an acid or alkaline condition of the secretion, unlike pepsin. Pepsin wears out; this does not. I believe this is quite a broad idea, and one worthy to be thought of, for just that condition of things would sometimes exist in canals that might be affected by the use of papoid.

*Dr. Sailer.*—Has the action of this papoid a tendency to destroy the sensitiveness of the pulp when it is in such a condition that we cannot touch it without causing severe pain to the patient?

*Dr. Howe.*—I cannot answer that question. I do not suppose any portion of pulp-tissue in which vitality lingered would be digested, but I should think that digestion and removal of the devitalized portion would assist materially the subsequent effort to devitalize the remaining part.

*Dr. Perry.*—I want to say a word in answer to Dr. Lord, not exactly in defence of Dr. Clapp, because I do not think he needs to be defended, but I think he may be misunderstood. Doubtless, as Dr. Davenport believes, there is some risk in the use of the oxyphosphate as a base for the gold, but there is some good in it, if carefully used, remembering always to use the crystal gold, as stated by Dr. Clapp. Perhaps that distinction has not always been made. I can easily see that other forms of gold could not succeed. I have many times started my fillings with a small portion of oxyphosphate in the bottom of the cavity, but I would never depend on the oxyphosphate for holding the gold in after it was placed. It would be only a momentary help for me in getting the filling started. In reference to the filling that was exhibited by Dr. Clapp and displaced by the thumb-nail, it must be remembered that Dr. Clapp stated that he had never put a filling in the mouth in that way. He has since sent me the same tooth refilled, and the filling seems very secure.

In reference to the use of the amalgam in the bottom of the cavity, Dr. Lord stated that he had not tried it; that he hardly thought it would be possible to combine the two, using the gold with and against the soft material. If the matrix is tied tightly around the tooth, so it does not yield to any great extent, and the amalgam is packed in the lower third, the excess of mercury taken off so that the amalgam in a few moments becomes practically hard, and then crystal gold used, I think it will be successful. I have heard several gentlemen say that they have not succeeded in the operation, and, upon being questioned, they stated that they had not used crystal gold. You cannot expect to succeed with foil, but with crystal or Steurer's gold it is an easy matter. I anneal the first pieces thoroughly, so they will take away all the excess of mercury, and then I reject those pieces of gold; then put on the pieces of crystal gold, hold them in place, and work them into the filling, having the matrix tied tightly by that beautiful method shown by Dr. Clapp, of tying it with thread or silk, and adapting it to the contour of the tooth; then the gold can be packed carefully until the undercuts are reached, so as to steady it, after which you can proceed rapidly. I have performed that operation very often since Dr. Clapp described it, and I have watched for an opportunity to say a word about it here before the Society. I am sure there is some good in it.

In reference to Dr. Lord's suggestion of packing the amalgam at the cervical wall after the gold has been packed, I think it a very good one. I have done that when I found defects in my filling after taking off the matrix. I sometimes pack soft gold in large masses at the cervical wall, expecting to make a condensation, and to add more gold along that border after the matrix is removed, and have had what I consider most excellent results, and I think Dr. Lord's suggestion of the use of amalgam in that way is most valuable, and will help many persons. I would not try to get an absolutely perfect margin in some cases at first, but I would take off the matrix and then do the best I could afterwards, either with gold or amalgam.

*Dr. Niles*, of Boston.—One can hardly help but agree with what Dr. Perry has said in regard to the packing of amalgam and gold together in certain cases. I have experimented with the process a little, and I find that we must be cautious as to the amalgam used, especially in cervical cavities. We all know that we possess but little information in regard to the manufacture of our amalgams as to the combination of metals used; while our fillings may be well placed and our work done perfectly, if the amalgam does not stay where it is placed, the filling will soon become worthless, no matter how nicely it has been executed. I have found a number of fillings recently where the amalgam bulged out very badly at the necks of the teeth and decay had begun; it was necessary to remove a number of fillings that had been so made by a very skilled dentist in our city. I also saw others in the same mouth doing well. Whether two different makes of amalgam had been used or not I do not know. The chief benefit of this process is, I believe, in that it expedites filling. The method which I adopted very early in practice, and which may be new to some of you, is to keep my gold cylinders exposed freely to the air some length of time before they are used. I find it changes the nature of the gold very much indeed, and it is my custom to keep my cylinders which I use first in packing in the cavity exposed to the air for at least three weeks or a month. They become very soft and tough, and in my method of packing, which I can do just as quickly as with amalgam, after the cavity is two-thirds filled, I use my cohesive gold for filling. It is done very quickly; and where the cavity is of comparatively easy access, there is no advantage in using amalgam. All of my amalgam is prepared from a formula arranged some time ago, so I know what to expect of it. It is good amalgam, and seems to stay where I put it, and yet I have not sufficient confidence to use it soft underneath the gold, unless in extreme necessity from certain conditions which I wish to overcome at the cervical margin.

*Dr. Littig.*—I should like the Secretary to read a resolution which I want to offer to the Society, and which they can take action upon or not, as they wish.

WHEREAS, The very high tariff duties on dental goods have for years been a serious hinderance to the dentists of this country in obtaining instruments and materials from abroad, and

WHEREAS, The last two tariff laws considerably increased the duties, and have effectually prevented the importation of many foreign products,[1] the privilege of using which would be a decided benefit to us and to our patients; therefore be it

---

[1] Some of which cannot be manufactured in this country.

*Resolved*, That we believe it to be desirable and expedient for the dentists throughout the United States to petition Congress to materially reduce the duties on all foreign dental goods. We believe that every dental society in the country would gladly unite in endorsing a general petition to this effect.

The Dental Protective Association of the United States has already rendered extraordinary service to all dentists, and through the self-denying labors of its chairman, Dr. J. N. Crouse, is still working to relieve us of onerous burdens.

As this Association is organized for such work, it seems appropriate that the effort to obtain a reduction of tariff duties should be made through this the largest and most influential dental organization in the country; therefore be it further

*Resolved*, That we request of Dr. Crouse that this work be undertaken by the Dental Protective Association, and that the Corresponding Secretary of this Society forward to Dr. Crouse a copy of these resolutions, making known to him the desire of this Society that he communicate to us his decision after due consideration.

*Dr. Littig.*—I should like free discussion on this subject, because I should not want it to go before the public without some endorsement which would be equal to the emergency.

*Dr. Perry.*—I think it is worthy of the notice of any society. The object of a dental society is to disseminate knowledge and benefit the dental profession at large. I move the adoption of the resolutions.

*Dr. Bogue.*—I do not like, under these circumstances, on the spur of the moment to suggest amendments in a paper that has evidently been prepared with a great deal of care, but Dr. Littig practically gives me permission to intimate that one point at least has not been noticed here that might have some bearing on the subject. It should be mentioned that some of the materials are not and cannot be manufactured in this country.

*Dr. Littig.*—I certainly will accept that amendment, as it is a very good one.

*Dr. Bogue.*—It is well known to the gentlemen present that the dental dealers took a great deal of trouble to see that that tariff was enacted, and no doubt they would fight this. It seems to me very becoming in this Society to take the initiative to bring a matter of this kind before the whole profession. I would like to suggest that Dr. Littig look over the first few lines and see whether that suggestion could not even be made a little stronger than I have stated.

*Dr. Bishop.*—I would suggest that this resolution be referred to Dr. Littig to report at the next meeting, with a list of the articles that we are paying more for by the increase of duties. I would like to know what they are.

*Dr. Littig.*—Everything we use that comes from the other side has been increased since the last tariff from ten to fifteen per cent. The reason my attention was called to it was that the teeth were introduced as porcelain products, and there was a dispute in reference to it. I was called upon to go before the commissioners and testify as to what the teeth were composed of. After giving my statement and having an analysis made of the teeth, they were introduced coming to this country as feldspar productions, which reduced the tariff on the English teeth. Now they are five per cent. more than they were before the passage of the McKinley bill. It is based upon a different thing altogether. It is based upon the cost of the article, instead of the *ad valorem* as it formerly was. It makes a difference in the amount of almost everything we get from abroad.

*The Chairman.*—The idea that Dr. Bogue has suggested has been added by the Secretary, and if he will kindly read it, I should like to know the pleasure of the gentlemen present in regard to it.

The Secretary then read the resolution as amended.

*Dr. Perry.*—Dr. Bogue said very justly that doubtless the interest of the dealers was enlisted to increase the duties. Their interest will be to keep those duties to the highest possible rate. How can we protect ourselves if not in this organized way?

*Dr. Sailer.*—The only point that presents itself to my mind is whether the resolutions are strong enough, and if it would not be better to refer it to a committee to report at our next meeting. It will be the starting-point of a great movement, and the starting-point should be as strong as possible.

*Dr. Perry.*—It seems to me that if this Society take the initiative in this way it would be sufficient. It will take proper course as it proceeds. The season is waning, and I have heard that there will be a revision of the tariff. A month lost may be of some importance. The question could be settled this evening.

*Dr. Howe.*—This is a resolution merely requesting the Dental Protective Association of the United States to undertake this work. If they undertake it, it will be their function to decide upon the way of doing it. The object of the resolution is that we, the New York Odontological Society, ask Dr. Crouse to undertake to obtain the petitions and get them presented to Congress. If we ask him now, we may get an answer before our next meeting. If for any reason he should see fit to decline, it would then be our province to discuss it further.

*Dr. Perry.*—Our next meeting is the last one of the season. It seems to me a great deal could be done during the summer, particularly in view of the Columbian celebration.

Motion carried.

Professor Frank Abbott then read the paper of the evening, entitled, "Bichloride of Mercury and Chloride of Zinc in the Treatment of Pulpless Teeth and Alveolar Abscess."

(For Dr. Abbott's paper, see page 561.)

DISCUSSION.

*Dr. Abbott.*—In view of some of the discussions which have taken place in reference to the disposition of dead or partially dead pulps in the roots of teeth, it is perhaps well for me to make the statement that I refer in this paper to nothing except absolutely dead and disintegrated pulp-tissue.

*Dr. Howe.*—It will be remembered by many here that Dr. C. T. Stockwell, of Springfield, has been an advocate of immediate root-filling, and on that point I wrote to him recently, asking him to come and discuss this question. He was unable to come, but he wrote me the following:

"The paper which I contributed to the discussion of this subject by the New York State Society in May, 1887, and published in the Transactions of the Society for that year, gives in sufficient detail not only the method of treatment I had then practised for several years but my present method also. Thus my experience for more than eight years does not, as far as I am personally concerned, suggest any radical change. The method has come to be known as that of immediate root-filling. This word 'immediate,' however, needs to be taken with a grain or two of allowance. Dr. Abbott does not, as I understand, fill roots with any material as the first step in the operation. He rather treats with bichloride of mercury in order to obtain certain conditions, after which he fills with oxychloride of zinc. So in my method I use $H_2O_2$ and bichloride of mercury in water until I secure certain conditions. When these conditions are gained I proceed to fill the roots with gutta-percha, moistened liberally with a solution of iodoform and eucalyptol (Sander & Sons' preparation). The same results would be had, I presume, if oxychloride of zinc or chloro-percha were used. The requisite condition to be gained before filling is, of course, 'the elimination of putrescible matter and the destruction of septic agents,' as was stated in the paper referred to, and my experience convinces me that this condition can be secured at one sitting in the great majority of cases. I may here say that I do not often fill at the first sitting cases that present soreness caused by pericemental inflammation. In regular practice there is seldom occasion for haste, and to be doubly sure is the safest course. It is exceedingly rare, however, that results by experimental fillings do not indicate that a permanent filling might not as well and as safely have been made. Of course there are a large number of cases where, for various reasons, it is difficult, to say the least, to gain ready access to the extreme apex of the canals of roots. This is often the case with the molars. In this class of teeth I have sometimes resorted to treatment with a strong solution of nitrate of silver, and then filled as best I could. So far my experimenting with this agent, barring the objectionable discoloring of the tooth, is all that could be desired. I have just sent a brief note relative to this matter to the INTERNATIONAL DENTAL JOURNAL, which, I presume, will appear in the June number. I have also, in a few cases, used Dr. Schreier's preparation of potassium and sodium, and so far am much pleased with the results. It may be said I get the same results practically that is had when the nitrate of silver is used, minus the discoloration of the teeth. I have yet to have my first case where the least indication of soreness or pericemental inflammation has followed when either of these two agents has been used, and the roots filled at the same sitting. Thorough work must, however, be done. One must know the condition he seeks to gain, and be able to recognize it when gained, else how can success be hoped for? Pure empiricism here, as elsewhere, will fall into deep pits more or less often."

*Dr. Perry.*—These two gentlemen must have a great deal of faith and hope, or they have struck a far-reaching and scientific fact. I have sometimes followed this treatment in the manner that has been spoken of, but always with great fear of causing trouble, and never to the extent of filling up more than the extreme apex of the root, leaving the subsequent operation of filling the canal and pulp-chamber to be performed some days after the test has been determined. I should not have the courage to go that far. I am much pleased to hear from both of these gentlemen in this way. It is certainly encouraging to us to find that they consider this treatment safe. It will be a great advantage to be able to control those teeth in that way, without the long treatment which we have used heretofore.

*Dr. Bogue.*—Neither the essayist nor the letter read tells us whether the cases under consideration are those which are ordinarily known as blind abscess,—that is, pericemental irritation,—or whether they are actually open abscesses, and it seems to me that it makes a vast difference. Dr. Hurd, whom every one remembers, got up one evening in Brooklyn, when a gentleman had been talking about what had happened in his practice, and said, "Mr. President, the gentleman has a great deal to say about 'my patients.' Mr. President, the gentleman forgets that the 'critters have legs, and they use 'em.'"

Two of those who have been most prominent as advocates of immediate root-filling are persons with whose practices I happen to be much interested and rather familiar. I do not refer to any one in New York. "That the critters have legs, and they use 'em," I do know, for I have seen many of them after treatment who refused to return to their operator, and as the result of the effort to treat what we know as blind abscess by immediate root-filling, I have seen cases so bad that I felt sympathy for both patient and operator.

I rise, therefore, to beg our essayist to make as distinctly as he is willing his position clear before us as to what he means, or *if* he means to apply his particular course of treatment to the roots of teeth having open abscesses only; I shall be very glad to hear of his admirable success. I can readily believe that he has had just that success. Can I accept his figures the same as I accept those of Dr. Cunningham, of England, who used Fowler's solution of arsenic. He had, according to his tables exhibited in Paris, but two or three failures, and all the rest were successes. If we are to undertake to syringe out a canal closed at the upper end, it seems to me there will be regurgitation, and if we undertake to inject remedies, and then fill, relying upon the syringing, what is the basis on which that treatment is to succeed? At present I do not quite understand.

*Dr. Abbott.*—I do not like to accuse Dr. Bogue of being asleep when anything interesting is being discussed, but I am afraid I must do so in this case. I do not recognize any such condition as what is termed "blind abscess," for no such thing exists. There is periosteal inflammation or irritation, but when we have an abscess pus is present, and there can be nothing blind about it. Now for the next point. In the first place, Dr. Stockwell says one wants to understand the condition that he has to deal with, and the nature and effect of the remedies that he uses. Then it is fair to presume that his treatment will be successful. This covers the ground. I have for twenty-five years treated abscesses in no other way than immediately to fill the roots after properly cleansing. I have never treated a tooth in my life and left it open to take care of itself, unless periosteal irritation was present. It will be remembered that twenty-five years ago I took issue with our late friend Dr. Atkinson upon this point, claiming that it was unnecessary to treat teeth in that way, and that it really prolonged the trouble.

*Dr. Perry.*—Does not everybody do that,—treat an abscess and fill the tooth at once?

*Dr. Abbott.*—Dr. Atkinson treated some cases for six months. He taught this method to every one he could, far and wide; and I presume—in fact, I know—that very many practitioners follow his methods in treating teeth *without* an abscess; and where there is no pericemental irritation, you will discover that, upon being opened, there is sometimes a very bad odor that penetrates everything. There is no abscess, however. If you disturb the living tissue at the end of the root, and leave it open, so that putrefying material can come in contact with it, or admit the air, it will, in combination with the heat and moisture already present, cause trouble. Suppose you destroy every organism in a pulp-canal and leave ptomaines behind, putrefaction will almost surely recur. These ptomaines are washed out or rendered inert by the bichloride of mercury. The bichloride of mercury seems to have the effect of destroying and getting rid of all putrefactive substances. To show you how much confidence I have in this work myself, I will say that, some four years ago, I had a tooth in my own mouth—an upper molar—which had been dallied with more or less until its pulp had died. One day I broke the side of it off. I went to a dentist, who is not present to-night, I am sorry to say, and he opened the tooth, and said, "Why, the pulp is dead." He opened the pulp-chamber and the canals. He could seemingly go to the very end of each of them. I do not think he used bichloride of mercury in the treatment of the case, but he washed them out with a syringe as best he could. He filled them under my directions, and put on the crown of amalgam. That tooth is in my head now, as sound as possible, and never gave me any trouble, except for an hour or two after the operation. He forced the material through one or two of the small roots, and the sensation, of course, was very unpleasant. That shows you the confidence I had myself at that time in this manner of treating these cases. There was no subsequent periosteal trouble. This work I do every day; sometimes I fill two or three roots of different teeth in the same mouth in one day. I have yet to find a case which was not successful, excepting where there has been an abscess previously; then there seems to be at the root of the tooth a condition which means inflammation, more or less severe, for a few days.

But that is the only kind of case that I have any trouble with. If you will follow the directions explicitly as given in the paper, I will guarantee that in nine cases out of ten you will have no difficulty.

*Dr. Perry.*—Do you make any attempt to dry out the canal with hot air?

*Dr. Abbott.*—No; I have the antiseptic in the canal, and I want it there. In dressing a wound, no surgeon would think of removing the antiseptic he has used in washing it before he put his dressing on. In order to intensify the antiseptic action I put bichloride of mercury, 1 to 2000, in the filling-material. I do not use the chloride of zinc alone, of course, in cases where there is no abscess.

*Dr. Bogue.*—Suppose a patient comes in with what he calls a sore tooth. On drilling into the tooth, you discover a dead pulp. What then?

*Dr. Abbott.*—I cleanse the canals thoroughly, then wash them out with bichloride of mercury, 1 to 10,000, put a bit of cotton in the cavity of the tooth to prevent food from getting in, and then paint the gum with the solution I mentioned in the paper, and dismiss the case for a day or two.

*Dr. Howe.*—I always like confidence that is the result of intelligent conviction. Dr. Abbott believes that, after a root is filled, there will be no occasion to remove the filling. I have for years filled roots with oxychloride of zinc, and believe that it is the best root-filling I know of; but sometimes I hear it said that it is wrong to fill the root of a tooth in such a manner that you cannot remove the material, as if it were to be expected that you would have to take the filling out. I think that is very rarely the case, and even in the few instances in my practice in which I have undertaken to, and finally succeeded in removing the filling in the root, I have found it a useless procedure. I think the way to succeed in filling roots of teeth is to do it when satisfactory conditions have been obtained. I think that the idea that controls a person who feels that he must put the filling in a root so it can be taken out is very likely to cause an imperfection in the work. I much prefer to proceed with the idea that when I fill a root I mean to have it stay there.

*Dr. Perry.*—I cannot agree with Dr. Howe. I do not see why a canal cannot be as well filled with the expectation of taking the filling out as otherwise. I think a little bit of oxychloride of zinc on one of those broaches which I described at the last meeting, with a piece of silk at the end of it to close the apex, and the rest of the canal filled either with oxychloride or chloro-percha, with a little gold point or wire put into it, and that little gold point so made that you can pull it out easily, is just as good as any filling that can be made, and you have the advantage of being able to take it out if you should need to. I will not admit that the expectation of some day removing the filling will lead one to make a poorer one.

*Dr. Niles.*—There is one condition that would make me careful about filling a dead tooth promptly, and that is where the process has gone so far that the foramen has become enlarged, or when there are bare, exposed portions of the cementum uncovered by death of the peridental membrane. During the past year I have found this condition of alveolar abscess rather frequently. The last case was an inferior sixth-year molar. A young medical student came to me, saying that he had a fungous growth on the side of his gum that he had been burning out with caustic. It could at once be seen that it was a fistula from an alveolar abscess. I opened into the tooth thoroughly, treated it antiseptically, and sealed it up temporarily, leaving it so it could be removed, as it seemed to be a specially aggravated case. In a few days I saw it, and the conditions were about the same as they were when he first came to me. I opened and cleansed it, used antiseptics, and treated it for some days, each time drying and sealing it, but I did not succeed in causing the fistula to heal. I finally said to the young man, "I fear that you will have to lose the tooth." He became impatient with the treatment, and requested that I extract it, with a possible prospect of replacing it, but I found both of the roots of the tooth denuded of the peridental membrane at least one-third of the length. Not only this, but it had become roughened by absorption, resembling an absorbed deciduous tooth. In cases of this kind, I know of no treatment that will heal the fistula. There is exposed foreign substance to which the tissues will not heal kindly, and will always be a source of irritation. I do not see how we can anticipate the conditions of any root The young man referred to told me the fistula had been of six months' standing. I could hardly credit that the tooth was living six months previous. Yet he assured me that such was the case. It was the most rapid absorption that I had ever seen.

*Dr. Ives.*—I want to ask Dr. Abbott what is the difference in the escharotic effect between carbolic acid and chloride of zinc. I have always used carbolic acid for a fistula, and I do not think I ever had to make the application twice.

*Dr. Abbott.*—Carbolic acid, according to my experience, becomes very much diluted with the liquids in the tooth and abscess. I object to carbolic acid on general principles, as I do to anything that is so disagreeable to have in the office. I am particularly sensitive myself in that direction, and I try in every way to keep rid of everything that has a disagreeable odor, so I use materials that will answer the purpose as well and are not so unpleasant. Chromic acid and other preparations have been used, but they will not do the work so kindly as the chloride of zinc. I think that will be admitted by everybody who has studied the subject. Using it as a weak solution around the roots of teeth, as an astringent, or a highly stimulating substance, it will do more work and do it better than any remedy I ever saw. You will say there is no occasion to use it if you cleanse your teeth thoroughly. This I admit. Dr. Niles has said that it was impossible to look at the roots of teeth when they are in the jaw. That is very true. We judge of the conditions, however, from the appearance of the abscess. An ordinary abscess can be treated and cured as easily as a wound in any other part of the body.

*Dr. Davenport.*—We usually notice in reading the endorsements of patent medicines that the different writers of such letters live in some out-of-the-way place where we cannot get at them. It has been much the same in the past regarding the advocates of immediate root-filling, for, with the exception of Dr. Stockwell and perhaps one or two other gentlemen who are well known, those advocates have not been readily accessible nor have they been men of scientific attainments. Feeling under great obligations to Dr. Abbott, I wish to thank him for his courage in giving us this result of his practice. We all know Dr. Abbott's position in the profession and in educational matters, and he would not jeopardize his reputation by advocating any extreme method, as this certainly is, without first having thoroughly proved it. For that reason I have great confidence in it, and feel that it is worthy of especial attention and study by us all.

*Dr. Bogue.*—Does Dr. Abbott make a difference in the treatment of upper and lower teeth?

*Dr. Abbott.*—None at all.

*Dr. Bogue.*—Upper teeth and lower incisors Dr. Perry has treated in the way described for many years. So have we all, probably; but lower back teeth most of us have been afraid to treat by that method. Even when an abscess existed, a fistula on a lower molar, it has presented conditions quite at variance with what might be called a similar condition in an upper tooth.

*Dr. Abbott.*—In a lower tooth the foramen is a depending opening; natural gravity takes any material left in the root of a lower tooth down to and through this foramen eventually, unless it is packed tightly. The roots of these teeth must be cleaned as thoroughly as the operator can do it. If that be done, he stands as good a chance to succeed as he does in the upper teeth. If the canals are made perfectly clean and treated as I have described, it makes no difference whether it is upper or lower.

*Dr. Perry.*—That question of cleanliness I advocated ten years ago, and I don't care how a man gets a root clean so long as he does so, whether by washing or by medicines. If your medicines are germ-killers, it makes little difference what you use.

A cordial vote of thanks was tendered to Dr. Abbott for his interesting paper.

Adjourned.

JOHN I. HART, D.D.S.,
*Editor New York Odontological Society.*

