# Correction

**Published:** 1893-10

**Authors:** E. A. Bogue


### Domestic Correspondence.

### CORRECTION.

AUGUST 23, 1893.

TO THE EDITOR:

SIR,—May I beg you to correct my remarks on page 593 of the August number of your journal? I quote from the report:

"It is generally understood by the gentlemen present that the dental dealers took a great deal of trouble to see that the clause covering dental goods in that tariff was enacted."

The S. S. White Dental Manufacturing Company assert positively that *they* did not use or try to use any influence with anybody with the object of affecting the rate of duty on a single article mentioned in the McKinley bill.

The president of that company informs me that he was opposed personally to any increase in the rate of duties.

E. A. BOGUE.

